# New Remedy for Asthma

**Published:** 1885-03

**Authors:** 


### A NEW REMEDY FOR ASTHMA.

A number of French observers have reported remarkably good results from the use of euphorbia pilulifera in asthma. An infusion is made by steeping one ounce of the fresh weed or one-half ounce of the dried plant in two quarts of water and reducing by simmering to one quart. The dose is a wine-glassful. Dr. Marsset reports nine cases where great benefit was obtained in organic or spasmodic dyspnœa by the use either of the above infusion, or of an aqueous extract of the Euphorbia. He recommends giving three or four wine-glassesful of the infusion after the evening meal. One of the patients who had been unable to work or even to lie down for months, was cured in a few days.

Experiments on animals show that the new drug is toxic in large doses. The symptoms and post-mortem appearances resemble those where death takes place from section of the pneumogastrics, and the drug is believed to act by modifying the vagus center of the medulla.

The active principle of the euphorbia appears to be a gum resin. soluble in water; no alkaloid has been discovered.—*Boston M. & S. Herald.*

